# Blood pressure demographics: nature or nurture ... ... genes or environment?

**DOI:** 10.1186/1741-7015-3-3

**Published:** 2005-01-07

**Authors:** Joseph Tomson, Gregory YH Lip

**Affiliations:** 1 University Department of Medicine, City Hospital, Birmingham B18 7QH, England, UK

**Keywords:** Hypertension, prevalence, genetic, environment, psychosocial factors

## Abstract

Hypertension is a growing worldwide problem associated with an increased risk of cardiovascular morbidity and mortality. However, the rates of prevalence of hypertension are higher in some populations than others. Although ethnic and genetic factors have been implied in the past to explain this, the environmental influence and psychosocial factors may play a more important role than is widely accepted. Examining the non-genetic influences in future hypertension research may be necessary in order to clearly define the local blood pressure demographics and the global hypertensive disease burden.

Hypertension is a common problem, with a consistent and continuous risk of cardiovascular disease and stroke associated with rising blood pressure levels [1]. Furthermore, effective treatment of blood pressures has been shown to cause reductions in morbidity and mortality from cardiovascular disease and stroke. The modern management of hypertension is even more complex, with the emergence of newer therapies, ageing populations and new clinical trial evidence, as well as the need for multiple agents to achieve target blood pressures, which are much lower than they used to be in the past [1].

The consequences of poor blood pressure control are huge. As high blood pressure is the most important risk factor for cardiovascular disease, it has been calculated that by achieving the target of 140 mmHg, there would be a reduction of 28–44% in stroke and 20–35% in ischaemic heart disease depending on the age. This would prevent approximately 21400 stroke deaths and 41400 ischaemic heart disease deaths each year – and these translate to approximately 42800 strokes and 82800 ischaemic heart diseases saved, making a total of 125600 events saved per year in the United Kingdom alone [2]. Even white coat hypertension is by no means a benign condition [3]. By 2020, the world population would be an estimated 7.8 billion people and hypertension currently is 'estimated' to affect about 1 billion worldwide – this figure will be rising. The growing numbers and the lack of concerted effort to tackle the burden of hypertension makes depressing reading.

Nonetheless, what is more intriguing and perhaps still not fully explained, is why some populations seem to have a much higher population prevalence of hypertension as compared to others. For instance, the prevalence and incidence of hypertension differs between the non-westernised and westernised populations. Even within the western world, the Afro-Caribbean or African-American black population has a higher prevalence of hypertension and target organ damage related to it, as compared to white Europeans or Americans [4]. Differences also exist within the same region, for example, with people of Eastern European origins having a higher prevalence of hypertension compared to elsewhere in Europe [5]. Understanding the reason(s) behind these geographical and ethnic differences would help devise effective measures in primary prevention.

Cooper et al [6], writing in *BMC Medicine*, address the issue of whether there is a truly genetic predisposition or perhaps an environmental influence is to blame for higher rates of prevalence of hypertension seen in some of these ethnic populations. In a well-designed pooled analysis, incorporating eight studies involving 8 white and 3 black populations from the North American, European and African populations – a dataset of nearly 85,000 patients – Cooper et al [6] examined patterns of blood pressure distribution in the different ethnic groups across three continents. They found a wide variation in hypertension prevalence among white and black racial groups, and the rates among blacks were not unusually high when viewed internationally. They therefore suggest that the impact of environmental factors among black and white populations may have been under-appreciated. Specifically, and perhaps contrary to expectations, the prevalence of hypertension was lower amongst the white peoples in Northern America and Canada, as compared to Europe.

Does this take us back to the drawing board? Perhaps environmental factors do play a more major role in developing hypertension than is widely accepted. Indeed, does urbanisation *per se *together with the unhealthy lifestyle and diet in the western world increase the risk of hypertension, compared to the rural, 'low stress', healthier lifestyle and dietary habits in Africa? Perhaps the genotype of black subjects was not idealised for the 'pro-hypertension' environment of the western world, leading to the greater risk of developing hypertension amongst blacks in the western world. This 'genetic predisposition' of certain ethnic groups, coupled with the 'wrong' environment, leads to an unhealthy combination that predisposes to cardiovascular disease [7]. However, the sociological definition of an ethnic group would be "people of the same race or nationality who share a common and distinctive culture", as it is impossible to consistently classify people by race. Genetic analyses have found more genetic variation within one ethnic group than between one group and another [8]. Therefore, race or ethnicity may appear to be more defined by customs, traditions, language and history than purely by genotype alone. Indeed, classification of race or ethnicity or skin colour, for example, is pretty subjective, imprecise and unreliable. Evidence for this exists in the differences in coronary risk factors in Indians, Pakistani and Bangladeshi populations in a British city, eventhough together these people might have been classed as 'Indo-Asian' but are clearly different [9]. Similarly, a Scottish Highland crofter is quite different from a Swede businessman, who would again be quite different from a Greek fisherman, although all would be ethnically classified as 'white caucasian'.

Can this 'wrong genotype in the wrong environment' hypothesis be applied to hypertension in black African-Americans? Efforts have already been made to understand the reasons behind the higher prevalence of hypertension in African Americans, with the underlying assumption that there may a genetically determined predisposition as compared to their white counterparts; however, no convincing data are available [10]. This may be because multiple genes determine human hypertension, at least in the vast majority of cases [11], and genetic factors have not been able to fully account for the differences in blood pressure prevalence between ethnic groups. Furthermore, elsewhere, black populations migrating to countries like UK have been shown to have similar blood pressures compared to the white UK population [12].

Numerous potential explanations for the higher prevalence of hypertension in blacks have been proposed. Genetic mechanisms have been used to explain familial aggregation of hypertension in Jamaican blacks and the intra-class correlation of systolic blood pressures amongst black twins [13,14]. Low renin levels noted in the USA black population have been hypothesised to be the result of a genetic 'maladaptation' which though benefited their earlier black ancestors to survive the torment of a transatlantic voyage under slavery, later turned out to be detrimental to survival due to the resultant avid salt retention [15]. Indeed, increased sodium sensitivity, abnormalities in sodium transport, increased vascular responsiveness to pressor stimuli, association between stresses of low socio-economic status and hypertension, and insulin resistance have also been suggested. Furthermore, ethnic differences in response to anti-hypertensive therapy are also well documented [4], with a potent effects of diuretics and calcium antagonists in black patients, compared to a relatively poor response to beta blockers and drugs that act on the renin-angiotensin system (such as the ACE inhibitors and angiotensin receptor blockers).

Notwithstanding the shortcomings of a retrospective, pooled study, with varying criteria for inclusion, and the intra-, inter- and cross-study observational errors, Cooper et al [6] have shown that the burden of hypertension seems to be more amongst the white population in Europe and that the global rates of hypertension amongst the black population are less in comparison. This study therefore sets the stage for a closer examination of data across geographic areas and calls for more stringent, standardised and possibly nationalised surveillance of blood pressure trends. If nothing else, the data suggests that inferences from cross-sectional studies done in certain geographic areas of a differing socio-economic stature cannot be extrapolated as logical benchmarks for other areas. Unintentionally perhaps, it beckons 'the scientist' to take a second look into the effect of other non-genetic mechanisms to explain the paradoxical findings. Surely more studies of a larger size are needed to confirm these implications.

Evidence that environmental and particularly psychosocial factors are important in the development of hypertension also comes from a series of epidemiological studies conducted in the early part of the last century, which have shown that urbanisation and adoption of a westernised life style leads to blood pressure rises. Many of these studies have been conducted in Africa, where the rural populace has a relatively low prevalence of hypertension. For example, primitive black populations living in more frugal circumstances in rural Africa have been shown to have low blood pressures. Evidence suggests that there are some populations that exist who are naïve to hypertension and related morbidity. Interestingly, their blood pressures hardly rise with increasing age, a common response to age in many other (urbanised?) communities [16-18]. The most important characterising feature of these populations were that, their lifestyle was traditional and they had not adopted or been under the influence of beliefs, customs or practices of another culture alien to theirs; the so-called 'unacculturated societies', with a close resemblance to the 'hunter-gatherer' lifestyle of primitive man. The other interesting feature observed was the constancy of electrolyte intake in the diet in these populations, which was in sharp contrast to the more 'developed' Western populations [19].

Migration, not withstanding the complexity of the studies in these populations, has been shown to significantly affect blood pressures. A study examining the migrant islanders into New Zealand showed raised population blood pressures, as well as an increased slope of the age-blood pressure relationship [20]. The Kenyan Luo migration study examined migration of rural tribes to the capital city Nairobi, also found that urbanised populations had higher mean blood pressures [21]. Chronic and excessive alcohol ingestion may also adversely affect blood pressure [22]. The relationship of hypertension with obesity has been demonstrated but weight loss seemed to have more pronounced blood pressure reductions in whites rather than blacks.

The issue of the influence of colour of the skin to blood pressures is even more complex. On the one hand, studies in America have shown relationship between the dark skin and blood pressures, leading to some suggesting that the link is genetic. In contrast, some argue that it is a manifestation of the stress and social pressure of having a dark skin that causes this. In Cuba, for example, where communist principles are considered to have broken the racial barriers, the ethnic differences in blood pressures were shown to be small, supporting the latter argument [23]. In addition, there may even be an effect of neighbourhoods or the social environment on blood pressure and cardiovascular disease [24,25].

Thus, the process by which a society becomes more economically advanced or "developed" seems to be associated closely with rates of hypertension prevalence. Indeed, lifestyle and dietary changes related to the so-called "development" seem linked to the prevalence of hypertension. In the INTERHEART population case-control study [26], which was an investigation into the association of psychosocial risk factors in patients with acute myocardial infarction, examining 11119 cases and 13648 controls from 52 countries, demonstrated higher prevalence of all four 'stress factors' (stress at home, at work, financial stress and major life events) in these patients, with consistency across regions, ethnicity and gender. Though data implicating stress as being contributory to the development of high blood pressures are limited and perhaps even hard to establish, a causal relationship between stress and developing high blood pressure does not appear to be an illogical assumption.

There remain many uncertainties to the relative importance and contribution of environmental versus genetic influences on the development of blood pressure – there is more than likely an influence from both. However, there is now evidence to necessitate increased attention in examining the non-genetic influences on blood pressure, a neglected area of hypertensive research but perhaps a goldmine for establishing causal influences. As stated earlier, the future of hypertension research should focus on more standardised and comparable protocols, with comparable designs in data collection. Multi-centric data collection with a view to establishing local or national blood pressure demographics is crucial for the formal assessment of the global hypertension burden and the implementation of cost-effective primary preventive measures.

## Pre-publication history

The pre-publication history for this paper can be accessed here:



## References

[B1] Willaims B, Poulter NR, Brown MJ, Davis M, McInnes GT, Potter JF, Sever PS, McG Thom S, British Hypertension Society (2004). Guidelines for management of hypertension: report of the fourth working party of the British Hypertension Society, 2004-BHS IV. J Hum Hypertens.

[B2] He FJ, MacGregor GA (2003). Cost of poor pressure control in the UK: 62000 unnecessary deaths per year. J Human Hypertens.

[B3] Gustavsen PH, Hoegholm A, Bang LE, Kristensen KS (2003). White coat hypertension is a cardiovascular risk factor: a 10-year follow-up study. J Hum Hypertens.

[B4] Lane DA, Lip GYH (2001). Ethnic differences in hypertension and blood pressure control in the UK. QJM.

[B5] Young JH, Parler P, Bristol B, Klag MJ The coming epidemic: hypertension in rural Kyrgyzstan, Central Asia. J Hum Hypertens.

[B6] Cooper RS, Wolf-Maier K, Luke A, Adeyemo A, Banegas JR, Forrester T, Giampaoli S, Joffres M, Kastarinen M, Primatesta P, Stegmayr B, Thamm M An International Comparative Study of Blood Pressure in Populations of European vs. African Descent. BMC Med.

[B7] Bhatnagar D, Anand IS, Durrington PN, Patel DJ, Wander GS, Mackness MI, Creed F, Tomenson B, Chandrashekhar Y, Winterbotham M Coronary risk factors in people from the Indian subcontinent living in west London and their siblings in India. Lancet.

[B8] Lewontin RC (1972). The apportionment of human diversity. Evol Biol.

[B9] Bhopal R, Unwin N, White M, Yallop J, Walker L, Alberti KG (1999). Heterogeneity of coronary heart disease risk factors in Indian, Pakistani, Bangladeshi, and European origin populations: cross sectional study. BMJ.

[B10] Cooper R, Rotimi C (1997). Hypertension in blacks. Am J Hypertens.

[B11] Garcia EA, Newhouse S, Caulfield MJ, Munroe PB (2003). Genes and hypertension. Curr Pharm Des.

[B12] Cruickshank JK, Jackson SHD, Beevers DG, Bannan LT, Beevers M, Stewart VL (1985). Similarity of blood pressure in blacks, whites and Asians in England: The Birmingham Factory study. J Hypertension.

[B13] Miall WE, Kass EH, Ling J, Stuart EL (1962). Factors influencing arterial blood pressures in the general population in Jamaica. BMJ.

[B14] Grim CE, Wilson TW, Nicholson GD, Hassel TA, Fraser HS, Grim CM, Wilson DM (1990). Blood pressure in blacks. Twin studies in Barbados. Hypertension.

[B15] Wilson TW, Grim CE (1991). Biohistory of slavery and blood pressure differences in blacks today. A hypothesis. Hypertension.

[B16] Poulter NR, Khaw KT, Hopwood BEC, Mugambi M, Peart WS, Rose G (1984). Blood pressure and associated factors in a rural Kenyan community. Hypertension.

[B17] Mann GV, Shafer Rd, Anderson RS, Stanstead HH (1964). Cardiovascular disease in the Masai. J Atherosclerosis Res.

[B18] Mann GV, Roels AA, Price DL, Merrill JM (1962). Cardiovascular disease in African pygmies. J Chron Dis.

[B19] Poulter N, Khaw KT, Hopwood BEC, Mugambi M, Peart WS, Sever PS (1984). Salt and blood pressure in various populations. J Cardiovasc Pharmacol.

[B20] Beaglehole R, Salmond CE, Hooper A, Huntsman J, Stanhope JM, Cassel JC, Prior IAM (1977). Blood pressure and social interaction and Tokelau and migrants in New Zealand. J Chron Dis.

[B21] Poulter NR, Khaw KT, Hopwood BE (1990). The Kenyan Luo migration study: Observations on the initiation of a rise in blood pressure. BMJ.

[B22] Lip GY, Beevers DG (1995). Alcohol, hypertension, coronary disease and stroke. Clin Exp Pharmacol Physiol.

[B23] Ordunez-Garcia PO, Espinosa-Brito AD, Cooper RS (1998). Hypertension in Cuba: Evidence of a narrow African-white difference. J Hum Hypertens.

[B24] Harburg E, Erfurt JC, Chape C (1973). Socioecological stressor areas and African-white blood pressure: Detroit. J Chronic Dis.

[B25] Diez-Roux AV, Nieto FJ, Muntaner C (1997). Neighbourhood environments and coronary heart disease: A multilevel analysis. Am J Epidemiol.

[B26] Rosengren A, Hawken S, Ounpuu S, Sliwa K, Zubaid M, Almahmeed WA, Blackett KN, Sitthi-amorn C, Sato H, Yusuf S, INTERHEART investigators (2004). Association of psychosocial risk factors with risk of acute myocardial infarction in 11119 cases and 13648 controls from 52 countries (the INTERHEART study): case-control study. Lancet.

